# The Contribution of IgG Glycosylation to Antibody-Dependent Cell-Mediated Cytotoxicity (ADCC) and Complement-Dependent Cytotoxicity (CDC) in Hashimoto’s Thyroiditis: An in Vitro Model of Thyroid Autoimmunity

**DOI:** 10.3390/biom10020171

**Published:** 2020-01-22

**Authors:** Marta Ząbczyńska, Katarzyna Polak, Kamila Kozłowska, Grzegorz Sokołowski, Ewa Pocheć

**Affiliations:** 1 Department of Glycoconjugate Biochemistry, Institute of Zoology and Biomedical Research, Faculty of Biology, Jagiellonian University, Gronostajowa 9, 30-387 Kraków, Poland; marta.zabczynska@doctoral.uj.edu.pl (M.Z.); kate.polak@uj.edu.pl (K.P.); kam.kozlowska93@gmail.com (K.K.); 2 Department of Endocrinology, University Hospital in Kraków, Kopernika 17, 31-501 Kraków, Poland; grzegsok@gmail.com

**Keywords:** IgG, *N*-glycosylation, sialylation, antibody-dependent cell-mediated cytotoxicity (ADCC), complement-dependent cyttoxicity (CDC), Hashimoto’s thyroiditis

## Abstract

Antibody-dependent cell-mediated cytotoxicity (ADCC) and complement-dependent cytotoxicity (CDC) are involved in destruction of thyroid tissue in Hashimoto’s thyroiditis (HT). *N*-glycosylation of the Fc fragment affects the effector functions of IgG by enhancing or suppressing the cytotoxicity effect. The aim of the present study was to assess the impact of HT-specific IgG glycosylation in ADCC and CDC, using in vitro models. The normal thyroid Nthy-ori 3-1 cell line and thyroid carcinoma FTC-133 cells were used as the target cells. Peripheral blood mononuclear cells (PBMCs) from healthy donors and the HL-60 human promyelotic leukemia cell line served as the effector cells. IgG was isolated from sera of HT and healthy donors and then treated with α2-3,6,8-neuraminidase to cut off sialic acids (SA) from *N*-glycans. We observed more intensive cytotoxicity in the presence of IgG from HT patients than in the presence of IgG from healthy donors. Removal of SA from IgG *N*-glycans increased ADCC intensity and reduced CDC. We conclude that the enhanced thyrocyte lysis resulted from the higher anti-TPO content in the whole IgG pool of HT donors and from altered IgG glycosylation in HT autoimmunity.

## 1. Introduction

Hashimoto’s thyroiditis (HT), an autoimmune thyroid disease (AITD), is characterized by infiltration of thyroid antigen-reactive T cells and by the presence of autoantibodies against thyroid antigens (thyroid peroxidase, TPO; thyroglobulin, Tg). Thyroid autoimmunity results in thyrocyte lysis and destruction of thyroid tissue, leading to hypothyroidism [1,2].

Studies of in vitro models have shown that the mechanisms of thyroid tissue damage in AITD rely on antibody-dependent cell-mediated cytotoxicity (ADCC) and complement-dependent cytotoxicity (CDC) triggered by autoantibodies characteristic for AITD. The role of autoantibodies against thyroid antigens in thyrocyte-destroying ADCC and CDC processes was demonstrated using in vitro models at the end of the last century. Thyroid cell lysis was significantly more intensive in the presence of HT sera than with healthy-subject sera in ADCC assays performed in vitro using peripheral blood lymphocytes from healthy donors as the effector cells. Lysis of thyroid target cells was also more severe when they were exposed in the absence of serum to effector cells isolated from the blood of HT patients, as compared with thyrocyte lysis in the presence of effector cells from healthy donors [3]. Rodient and coworkers showed that anti-TPO from thyroiditis sera, but not anti-Tg, participates in ADCC of porcine thyrocytes in primary culture. Thyrocyte lysis triggered by anti-TPO- and anti-Tg-depleted IgG was significantly decreased in comparison to cell death in the presence of the total serum IgG pool [4]. A study of an in vitro model consisting of primary human thyroid cells used as the target cells, serum samples from HT, Graves’ disease (GD) and primary myxoedema donors used as autoantibody sources, and PBMC serving as the effector cells confirmed the significance of thyroid-specific antibodies in the ADCC process. Thyrocyte lysis was significantly higher for each of the three patient groups versus control sera, and HT-specific antibodies were more effective than GD autoantibodies in triggering thyroid cell lysis [5]. The involvement of anti-TPO in CDC has also been shown in vitro. HT IgGs exerted the cytotoxic effect of the complement more efficiently than control antibodies did. Pre-incubation of HT IgG with purified TPO significantly decreased specific cell lysis, demonstrating that CDC is mediated by anti-TPO IgG. This effect was not observed in CDC with the use of HT IgG pre-incubated with purified Tg [6]. Rebuffat and coworkers confirmed the importance of anti-TPO in thyroid destruction, also using in vitro models of ADCC and CDC. Thyroid primary cells lysed in a process initiated by anti-TPO and effected by PBMC or HL-60 and THP-1 human monocyte cell lines. Highly effective lysis of thyrocytes by CDC was also observed when the cells were incubated with anti-TPO followed by guinea pig serum used as complement source [7].

IgG mediates an effector function via the ability to interact with its Fcγ receptor (FcγR) expressed on effector cells and with a C1q complement protein. It is well established that *N*-glycans attached to IgG Fc greatly contribute to the regulation of IgG effector functions [8]. IgG could induce pro- or anti-inflammatory signals, depending on the sugar composition of Fc *N*-glycans. Low core-fucosylation of Fc *N*-glycan enhances ADCC [9]. In general, terminal sialylation is responsible for anti-inflammatory activity, but the final effect of IgG sialylation depends on the monosaccharide composition of Fc *N*-glycans [10]. Modifications of Fc *N*-glycan structures are commonly used in therapeutic antibodies to modulate the efficiency of Fc binding to its receptor and to increase the effectiveness of therapy [11]. Changes of IgG *N*-glycosylation have been noted in different chronic diseases, including cancers [12,13] and inflammatory disorders [14,15,16,17]. Although analysis of IgG glycosylation has become a standard approach in work aimed at discovering new plasma biomarkers, functional analysis of altered IgG glycosylation remains largely neglected. Previously we demonstrated the altered *N*-glycosylation profile of IgG from Hashimoto’s thyroiditis patients. We observed significantly lower IgG core fucosylation in HT than in healthy individuals [18]. Analysis of IgG-depleted sera from HT donors indicated an increase of α2,3-sialylated di- and tri-antennary complex-type *N*-glycans on serum proteins [19].

**Figure 1:**
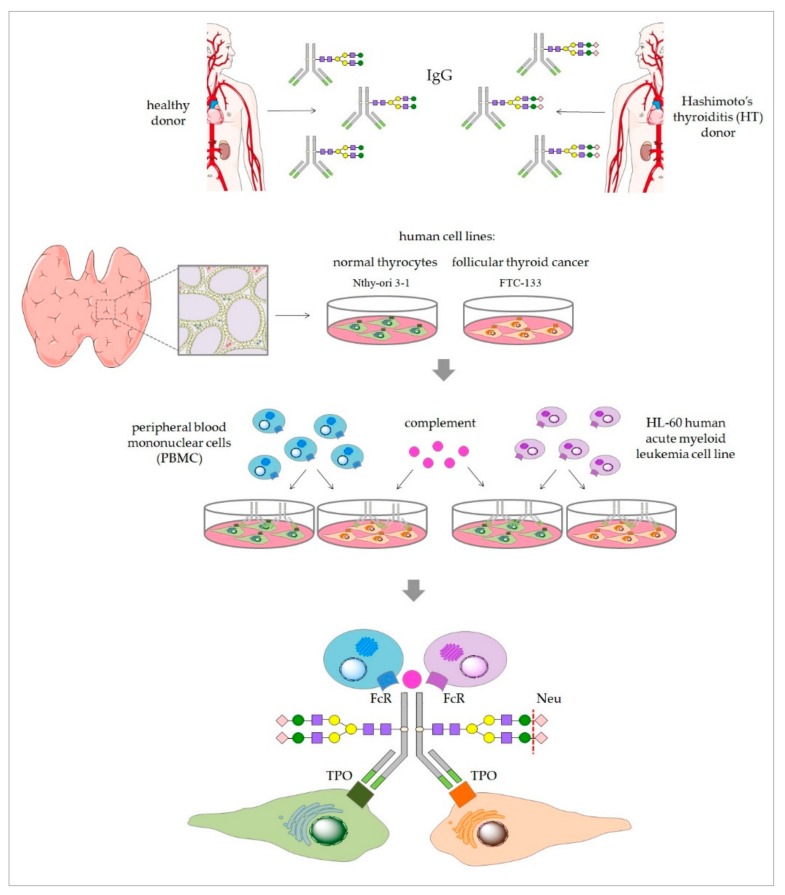
Scheme of antibody-dependent cell-mediated cytotoxicity (ADCC) and complement-dependent cytotoxicity (CDC) models and experiment stages. IgGs were isolated from Hashimoto’s thyroiditis (HT) and healthy donor (C, control) sera and treated with neuraminidase (Neu) to remove sialic acid from *N*-glycans. The key components involved in ADCC and CDC are the target cells expressing an autoantigen (thyroperoxidase, TPO), IgGs (isolated from HT and C donors) containing thyrocyte-antigen-specific anti-TPO, and effector cells with a surface receptor for IgG crystallizable fragment Fc (FcR) in the ADCC model and the complement in the CDC model. Nthy-ori 3-1 and FTC-133 cell lines were used as the target cells; PBMC isolated from healthy donors and the HL-60 cell line were used as the effector cells. Intact and Neu-treated IgGs from HT and the control were used as a variable element triggering ADCC and CDC.

Based on these recent studies, we hypothesized that altered *N*-glycosylation of serum proteins affects pathological processes occurring in HT, including thyroid destruction in ADCC and CDC processes. The present study is a functional analysis of differentially *N*-glycosylated IgG isolated from HT patients and healthy donors (control group) in in vitro models of ADCC and CDC. Normal and cancerous thyroid cell lines were used as target cells in both cytotoxicity models. Peripheral blood mononuclear cells (PBMCs) and the HL-60 cell line served as the effector cells in the ADCC model, and normal serum as an effector component in the CDC assay. We used cytotoxic assays (Figure 1) in a comparative analysis of thyroid cell lysis triggered by control and HT IgG, both enzymatically desialylated.

## 2. Materials and Methods

### 2.1. Ethical Statement

The study was conducted in accordance with the Helsinki Declaration and the protocol was approved by the Bioethics Committee of Jagiellonian University (Permit No. 1072.6120.145.2017). All blood donors gave their informed consent for inclusion before their participation in the study.

### 2.2. IgG Isolation

Blood samples were collected from HT female patients of the Endocrinology Clinic of the University Hospital in Kraków and from age- and sex-matched healthy individuals (control group). All donors were recruited to the study based on serum levels of TSH, anti-TPO and anti-Tg, and on thyroid ultrasonography. Blood was collected via venous puncture into clotting activator tubes (S-Monovette, Sarstedt), left for 5 h at RT for blood coagulation, and centrifuged (2500 rpm, 10 min, RT). Serum samples were kept stored under deep freezing until IgG isolation or CDC assay. IgG was isolated using protein G affinity chromatography. Serum samples (100 µL) from healthy donors (*n* = 24) and HT patients (*n* = 24) were diluted 1:1 with phosphate buffer (0.1 M sodium phosphate, 0.15 M sodium chloride, pH 7.5) and applied to a Protein G Spin Plate (Thermo Scientific, Waltham, MA USA; 45204). After repeated washing with the phosphate buffer, IgG was eluted with 0.1 M glycine (pH 2.5) and immediately neutralized with 1 M Tris (pH 9). IgG samples purified from the sera of 24 donors were pooled for further procedures.

### 2.3. Desialylation of IgG

Before desialylation, IgG eluted in glycine buffer was subjected to buffer exchange for PBS (Sigma-Aldrich, St. Louis, MO, USA; P4417), using Amicon Ultra 10 kDa filters (Millipore, Burlington, MA, USA). Then, α2-3,6,8-neuraminidase (Neu) from *Clostridium perfringens* (New England BioLabs, Ipswich, MA, USA; P0720) was used to remove sialic acid (SA) from IgG *N*-glycans. IgG (100 µg) was incubated with 200 U Neu in 40 µL of 50 mM sodium acetate containing 5 mM CaCl_2_ (pH 5.5) and incubated overnight at 37 °C. After desialylation, Neu was removed using VivaSpin filters (Sartorius, Göttingen, Germany), with a molecular weight cut-off of 50 kDa. The efficiency of desialylation was monitored using *Sambucus nigra* agglutinin (SNA) in lectin blotting (see Section 2.8).

### 2.4. Cell Lines and Culture Condition

The Nthy-ori 3-1 human thyroid follicular epithelial cell line and FTC-133 human follicular thyroid cancer cell line were kindly provided by Prof. Barbara Czarnocka of the Centre of Postgraduate Medical Education in Warsaw and by Prof. Anna Krześlak of the University of Łódź, respectively. Human acute myeloid leukemia HL-60 cell line was obtained from Dr. Małgorzata Opydo-Chanek of the Jagiellonian University. The cells used to the experiments were mycoplasma-free, as routinely determined by a chemiluminescence test (Lonza, Basel, Switzerland) and verified by PCR (forward primer: 5′-ACTCCTACGGGAGGCAGCAGTA-3′, reverse primer: 5′-TGCACCATCTGTCACTCTGTTAACCTC-3′, Oligo). The use of the Nthy-ori 3-1 cell line was reported to the Ministry of the Environment, because Nthy-ori 3-1 was qualified as a genetically modified microorganism (GMM).

Nthy-ori 3-1 and HL-60 were maintained in RPMI 1640 medium (Lonza, Basel, Switzerland; BE12-702F) supplemented with 10% FBS (Gibco, Paisley, UK; 10270-106) and antibiotics (100 units/mL of penicillin and 100 μg/mL of streptomycin, Sigma-Aldrich, P4333). FTC-133 was cultured in DMEM (Sigma-Aldrich, St. Louis, MO, USA; D5671) supplemented with 10% FBS and antibiotics. The cells were kept in a humidified incubator with 95% air and 5% CO_2_ at 37 °C. After confluence, Nthy-ori 3-1 and FTC-133 cells were subdivided into new flasks when ~80% confluence was reached. HL-60 cells were passaged after reaching cell density of 25 × 10^4^ cells/mL.

### 2.5. RT-qPCR

TPO mRNA expression in thyroid cell lines was determined by isolation of total cellular RNA using an RNeasy Plus Mini Kit (Qiagen, Hilden, Germany) and reverse-transcribed into cDNA using a High-Capacity RNA-to-cDNA Kit (Applied Biosystems, Foster City, CA, USA). cDNA was subjected to real-time quantitative reverse transcription PCR (RT-qPCR) using Power SYBR Green PCR Master Mix (Applied Biosystems, Foster City, CA, USA). The following primers were used for the TPO gene: 5′-TTGTACAACGGGTTCCCACT-3′ (forward) and 5′-GGAGGTCAGAATAGCGGTCA-3′ (reverse); and for the 18S rRNA reference gene: 5′-CCAGTAAGTGCGGGTCATAAG-3′ (forward) and 5′-CCATCCAATCGGTAGTAGCG-3′ (reverse). RT-qPCR data were quantified by the 2^−ΔΔCt^ method. The experiment was performed in triplicate.

### 2.6. SDS-PAGE and Immunodetection of TPO

Total cell extracts were obtained using RIPA buffer (Thermo Fisher Scientific, Waltham, MA USA) containing a protease inhibitor cocktail (Sigma-Aldrich, St. Louis, MO, USA; P8340). Equal amounts (25 µg) of Nthy-ori 3-1 and FTC-133 cell lysate proteins were separated under reduced conditions in 10% SDS-PAGE. Then the resolved proteins were electrotransferred onto a PVDF membrane (Millipore, Burlington, MA, USA) and incubated overnight in 2% BSA at 4 °C. The PVDF membranes were incubated for 1 h at RT with primary antibody anti-TPO (abcam, Cambridge, UK; ab203340) diluted 1:1000 in 2% BSA, and anti-GAPDH (Sigma-Aldrich, G9545) diluted 1:4000 in 2% BSA. After washing, the alkaline phosphatase (AP)-conjugated secondary antibody was used (diluted 1:4000): anti-mouse (Sigma-Aldrich, A2682) for TPO detection and anti-rabbit (Millipore, Burlington, MA, USA; AP304A) for GAPDH. The specific protein bands were visualized by colorimetric reaction after adding the substrates for AP: 5-bromo-4-chloro-3-indolyl phosphate (BCIP) and nitro blue tetrazolium (NBT) (Roche, Mannheim, Germany). The relative protein expression was quantified densitometrically using ImageLab software (Bio-Rad, Hercules, CA, USA). The experiment was performed in duplicate.

### 2.7. Flow Cytometry

The thyroid cells (5 × 10^4^) were incubated with anti-TPO primary antibody (abcam, ab203340) diluted 1:200 in 50 µL PBS for 45 min at 4 °C. After washing in PBS and centrifugation (1200 rpm, 10 min, 4 °C), the cells were incubated with AlexaFluor488-conjugated anti-rabbit secondary antibody (Invitrogen, Paisley, UK; A21206) for 45 min at 4 °C in darkness. TPO-stained cells were analyzed quantitatively with a FACSCalibur flow cytometer (BD Biosciences, San Diego, CA, USA) using CellQuestPro software (BD Bioscience, San Diego, CA, USA). The experiment was performed in triplicate.

### 2.8. Lectin Blotting

Lectin staining was performed as previously described [19]. *Sambucus nigra* agglutinin (SNA) specific for α2,6-linked sialic acid was used to determine the efficiency of IgG desialylation. Desialylated and untreated IgG (1 µg) from healthy donors and HT samples were separated on 10% SDS-PAGE stain free gels (Bio-Rad) in reducing conditions, followed by the electrotransfer onto a PVDF membrane (Millipore). The membranes were blocked with Carbo Free Blocking Solution (Vector Lab., Burlingame, CA, USA), overnight at 4 °C and incubated with biotinylated SNA (Vector Lab., B1305) diluted 1:4000 in TBS with ions (50 mM Tris-HCl, 150 mM NaCl, 1 mM MgCl_2_, 1 mM CaCl_2_, 1 mM MnCl_2_, pH 7.5) for 1 h at RT. After washing in TBS, AP-conjugated ExtrAvidin (Sigma-Aldrich, E2636) diluted 1:4000 in TBS was applied. α2,6-sialylated IgG chains were visualized in AP colorimetric reaction as described above (Section 2.6).

### 2.9. PBMC Isolation

Blood samples were obtained from healthy individuals. Blood was collected via venous puncture into EDTA collection tubes (S-Monovette, Sarstedt, Nümbrecht, Germany) on the day of the experiment. PBMCs were isolated by density gradient centrifugation using a Histopaque-1077 (Sigma-Aldrich, St. Louis, MO, USA; 10771). After washing twice in PBS, PBMCs were counted and resuspended in the appropriate culture medium.

### 2.10. ADCC Assay

Nthy-ori 3-1 and FTC-133 serving as target cells were harvested and seeded at a density of 1×10^4^ cells per well into a 96-well black clear bottom plate a day before the experiment. On the day of the assay, the medium was removed, the cells were washed twice with PBS, and 50 µL of the appropriate fresh medium supplemented with 10% ultra-low IgG FBS (Gibco, Paisley, UK; A3381901) was added to each well. Desialylated and untreated IgG (30 µg) in 15 µL of PBS was added to the target cells and incubated for 2 h in a CO_2_ incubator at 37 °C. Then, PBMC and HL-60 effector cells resuspended in 35 µL of assay medium were added in the following proportions of target to effector cells: 1:50 for PBMC and 1:12 for HL-60. After 4 h of incubation in a CO_2_ incubator at 37 °C, target cell lysis was measured by detection of DNA released from the dead cells into the medium (CellTox Green Cytotoxicity Assay, Promega, Madison, WI, USA; G8742) according to the manufacturer’s instructions. Fluorescence was measured at 485 nm Ex/535 nm Em using an Infinite F200Pro plate reader (Tecan, Männedorf, Zürich, Switzerland). The experiment was performed in triplicate. A set of controls of spontaneous cell lysis was used, including target and effector cells without IgG, target cells and IgG without effector cells, only target cells, and only effector cells. Maximum cell lysis of both thyroid cell lines was measured after application of lysis solution supplied with the CellTox Green Cytotoxicity Assay.

### 2.11. CDC Assay

Nthy-ori 3-1 or FTC-133 target cells (1 × 10^4^/well) were seeded into a 96-well black clear bottom plate on the day before the experiment. On the day of the assay, 50 µL of the appropriate fresh medium without FBS was added to the target cells after removing the medium and washing twice with PBS. Desialylated and untreated IgG (30 µg) in 15 µL of PBS was added to the target cells and incubated for 2 h in a CO_2_ incubator at 37 °C. Normal human serum was used as complement source in the CDC reaction. Human serum at a final concentration of 10% or 25% (*v*/*v*) in culture medium was added to each well. After 4 h of incubation, thyroid cell lysis was determined using the CellTox Green Cytotoxicity Assay (Promega, Madison, WI, USA) as described above (Section 2.10). CDC was repeated in four independent experiments.

### 2.12. Statistical Analysis

The expression of TPO in Nthy-ori 3-1 and FTC-133 cells was compared statistically using Student’s t-test. The differences in thyroid cell lysis triggered by control and HT IgGs in the ADCC model was analyzed statistically by two-way ANOVA followed by Tukey’s post hoc test using donor status (healthy or HT) and effector cell type (PBMC, HL-60) as the categorical variables. To compare the results obtained in the CDC model, two-way-ANOVA followed by Tukey’s post hoc test was applied. All of the statistical analyses were performed with Statistica 13.0 software (TIBCO Software, Palo Alto, CA, USA). *p* values < 0.05 were considered significant. The results are given as means of three or four independent experiments ± SD.

## 3. Results

### 3.1. TPO Expression in Nthy-ori 3-1 and FTC-133 Target Cells

**Figure 2:**
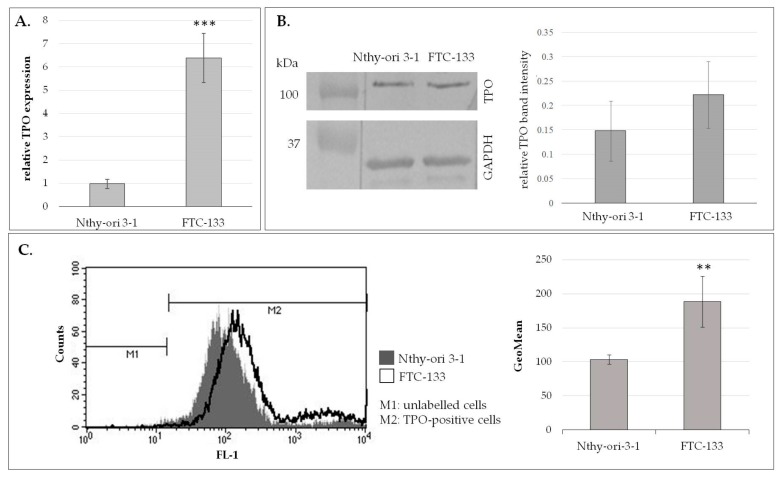
TPO expression in Nthy-ori 3-1 and FTC-133 target cells. (**A**) TPO mRNA level normalized to 18S rRNA reference gene. (**B**) Immunodetection of TPO (left panel) and the intensity of TPO bands (right panel). The protein extracts were separated on 10% SDS-PAGE, blotted onto a PVDF membrane, and probed with the specific antibodies against TPO and GAPDH (loading control). Molecular mass was verified using the protein standard (Bio-Rad). The intensity of TPO bands was measured densitometrically and normalized to the corresponding GAPDH bands. (**C**) Flow cytometric analysis of TPO surface expression. The representative histogram plot (left panel) shows TPO-positive cells (M2) determined in relation to TPO-negative cells (M1). The bar graph (right panel) presents geometric mean fluorescence (GeoMean). The data are shown as means ± SD obtained from three independent experiments. The difference between the target cells was determined statistically by Student’s t-test, with *p* ≤ 0.05 considered statistically significant; **p ≤ 0.01, ***p ≤ 0.001.

Of the two major autoantigens in HT, only TPO is expressed on the surface of thyrocytes [7] and can induce an immune response in the established in vitro models of ADCC and CDC. Before the use of Nthy-ori 3-1 normal thyrocytes and FTC-133 thyroid cancer line as target cells in the ADCC and CDC models, TPO expression was determined on mRNA and protein levels in both cell lines. RT-qPCR analysis showed a significantly higher amount of TPO mRNA in FTC-133 cells than in Nthy-ori 3-1 (*p* < 0.01; Figure 2A). The presence of TPO protein was shown by Western blotting in both cell lysates (Figure 2B) and by flow cytometry on the surface of both cell lines (Figure 2C). TPO surface expression was significantly higher in the FTC-133 cell line (*p =* 0.03; Figure 2C), raising the question of whether the expected cytotoxic effect in the planned ADCC and CDC assays will depend on TPO surface expression.

### 3.2. IgG Desialylation

**Figure 3:**
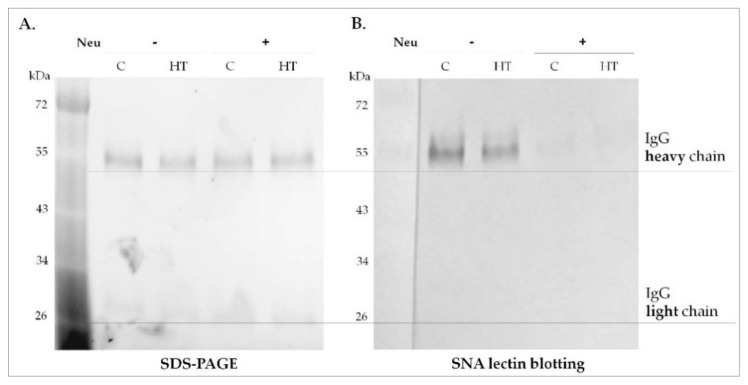
The efficiency of IgG desialylation. IgG samples from healthy donors (C, control) and Hashimoto’s thyroiditis (HT) patients after Neu treatment (+) and untreated (−) were resolved by SDS-PAGE in reducing conditions (**A**) and probed with *Sambucus nigra* agglutinin (SNA)-specific α2,6-linked sialic acid (**B**). Molecular mass of IgG heavy and light chains was verified using a PageRuler Prestained Protein Ladder (Thermo Scientific).

The significantly higher concentration of anti-TPO in the blood of HT patients than in that of healthy donors was determined by electrochemiluminescence assay. The characteristics of donors, including anti-TPO level, are presented in Table 1. The whole pool of serum IgG isolated from HT patients and healthy donors was desialylated by Neu with broad specificity to α2-3,6,8-linked sialic acid. The efficiency of enzymatic desialylation was verified by lectin blotting with SNA specific for α2,6-linked SA, because α2,6-sialylated *N*-glycans are present on IgG much more frequently than are oligosaccharides with α2,3-SA [20]. The absence of an SNA staining signal in Neu-treated IgG from both HT and control samples confirmed the efficiency of IgG desialylation (Figure 3). Before using IgG in the ADCC and CDC assays, Neu (molecular weight 43 kDa) was removed from it by centrifugation on a VivaSpin filter with a 50 kDa cut-off.

### 3.3. Comparison of Thyrocyte Lysis Triggered by IgG from HT and Healthy Donors in ADCC and CDC Models

Based on previous results [4,6,7,21], we assumed that anti-TPO in the whole pool of IgG would contribute to the cytotoxic effect on thyroid cells, mediated by immune effector cells (ADCC) and the complement (CDC). For both in vitro assays, we used the same amount of IgG from HT and healthy donors (30 μg) per well, but these IgG samples significantly differed in anti-TPO concentration (Table 1) and in their glycosylation profile [18]. Nthy-ori 3-1 and FTC-133 human thyroid cell lines served as the target cells in both models, and the HL-60 human cell line and freshly isolated PBMC were used as the effector cells in the ADCC assay. The optimal amounts of model components adapted to 96-well microplate format, including ratio of target to effector cells, IgG concentration and incubation times, were selected based on literature data [4,7] or were experimentally established (volumes of cell and IgG suspensions). To evaluate the lysis of target thyroid cells triggered by IgG from HT and healthy donors, we compared the signal intensity of the fluorescently labeled DNA released from dead cells in the CellTox Green Cytotoxicity Assay (Promega).

**Figure 4:**
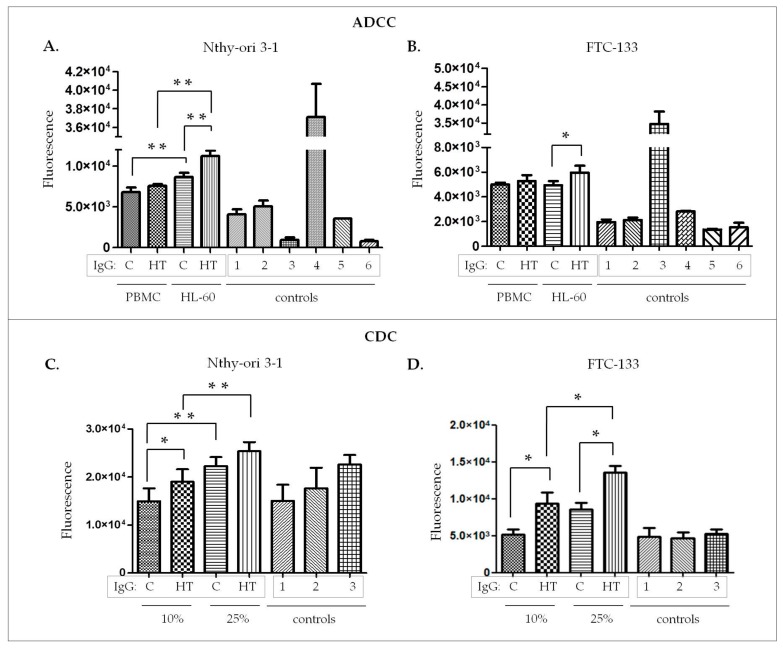
Nthy-ori 3-1 and FTC-133 target cell death, expressed as fluorescence intensity determined in ADCC and CDC models. (**A**) Nthy-ori 3-1 and (**B**) FTC-133 cell lysis triggered by IgG isolated from Hashimoto’s thyroiditis patients (HT) and healthy donors (**C**, control) and mediated by peripheral blood mononuclear cells (PBMC) and the HL-60 human promyelotic leukemia cell line in the ADCC model. (**C**) Nthy-ori 3-1 and (**D**) FTC-133 cell lysis triggered by C and HT IgG in the presence of 10% and 25% serum from healthy donors in the CDC model. In each experiment, a set of controls was prepared. The following controls were used: in the ADCC model: (1) target cells incubated with C IgG, (2) target cells incubated with HT IgG, (3) untreated target cells, (4) whole target cells lysed, (5) HL-60 cells, (6) PBMC; in the CDC model: (1) target cells, (2) target cells incubated with 10% serum, (3) target cells incubated with 25% serum. The data are shown as means ± SD. The differences between the experimental variants were analyzed statistically by two-way ANOVA with the post hoc Tukey test (*p ≤ 0.05, **p ≤ 0.01).

We found significant differences in thyrocyte lysis between HT and control IgG in ADCC (Figure 4A,B) and CDC (Figure 4C,D) assays. In the ADCC model, there were statistically significant increases of Nthy-ori 3-1 (*p =* 0.0018; Figure 4A) and FTC-133 (*p =* 0.04; Figure 4B) cell lysis induced by HT IgG in the variant with HL-60 as the effector cells. We did not find any differences in the lysis of both target cell lines between HT and control IgG in the presence of PBMC effector cells (Figure 4A,B). Nthy-ori 3-1 lysis was more efficient in the presence of HL-60 cells used as the effector cells than in the presence of PBMC (*p =* 0.004 for control IgG, *p =* 0.001 for HT IgG; Figure 4A).

In CDC assays, healthy-volunteer sera at 10% and 25% final concentration were used as complement source. IgG concentration and the incubation times were the same as in the ADCC assay. Analysis of target cell death under the action of the complement showed significantly higher thyroid cell lysis induced by HT IgG in the presence of 10% sera in the case of Nthy-ori 3-1 (Figure 4C), and for both serum concentrations in FTC-133 (Figure 4D). As expected, the cytotoxicity effect was stronger at the higher serum concentration (25%) in both target cell lines (Figure 4C,D).

### 3.4. The Contribution of IgG Glycosylation to Thyrocyte Lysis in the Cytotoxicity Models

The enhanced cytotoxicity in the presence of IgG from HT donors (Figure 4) resulted from higher anti-TPO content in the whole IgG pool (Table 1) used in both cytotoxicity assays. The last part of our study was designed to assess the contribution of IgG glycosylation, which is altered in HT autoimmunity [18], in thyroid destruction. To analyze the functional effect of IgG with altered *N*-glycosylation in HT versus control donors, we performed ADCC and CDC cytotoxic assays using enzymatically desialylated IgG. We express the cytotoxic effect as the percentage of thyrocyte lysis (% cell death), according to the following formula: (fluorescence of desialylated IgG/mean fluorescence of untreated IgG) × 100%.

**Figure 5:**
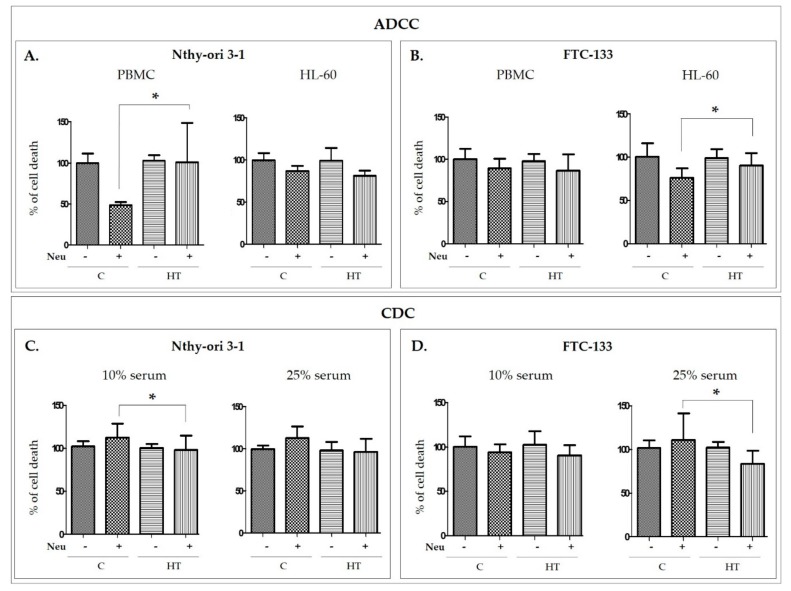
Percentage of Nthy-ori 3-1 and FTC-133 target cell death induced by α2-3,6,8-neuraminidase (Neu)-treated (desialylated) and intact IgG in ADCC and CDC models. Relative (**A**) Nthy-ori 3-1 and (**B**) FTC-133 cell lysis triggered by desialylated IgG (+) and untreated (−) isolated from healthy donors (**C**, control) and Hashimoto’s thyroiditis patients (HT) and mediated by peripheral blood mononuclear cells (PBMC) and the HL-60 human promyelotic leukemia cell line in the ADCC model. Relative (**C**) Nthy-ori 3-1 and (**D**) FTC-133 cell lysis triggered by desialylated IgG (+) and untreated (−) from C and HT donors and mediated by 10% and 25% serum from healthy donors in CDC model. The bar graphs represent the percentage of cell lysis triggered by desialylated IgG (Neu+) versus untreated IgG (Neu−). The data are shown as means ± SD. The differences between the experimental variants were analyzed statistically by two-way ANOVA with the post hoc Tukey test (*p ≤ 0.05).

Statistical analysis showed significantly higher Nthy-ori 3-1 cell death caused by PBMC in the presence of desialylated IgG from HT than in the presence of control IgG (*p =* 0.021; Figure 5A). More efficient lysis in the ADCC process was also found for FTC-133 cells treated with desialylated HT IgG in comparison to control IgG without SA; for thyroid cancer cells, the difference was observed in the treatment in which the HL-60 line used as effector cells (*p =* 0.021; Figure 5B).

An impact of IgG desialylation on thyrocyte lysis was also observed in the CDC assay, but the difference between HT and control IgG was the opposite (Figure 5C,D) of the changes obtained in ADCC (Figure 5A,B). Nthy-ori 3-1 cell death in the presence of 10% serum was less intensive for desialylated IgG from HT than for control IgG (*p =* 0.014; Figure 5C). A weaker cytotoxic effect for HT IgG than for control antibodies was also found in FTC-133 treated with 25% serum (*p =* 0.033; Figure 5D).

The differences in the ADCC and CDC cytotoxicity effects after removal of sialic acids from IgG isolated from HT and healthy donor sera demonstrate that altered glycosylation in HT contributes to the intensity of thyrocyte destruction in the established models.

## 4. Discussion

The main purpose of the present research was to evaluate the possible effect of IgG *N*-glycosylation on its effector functions in thyroid cell lysis, using established in vitro ADCC and CDC models of autoimmune thyroid destruction occurring in HT. We recently described the changes in IgG *N*-glycosylation characteristic for AITD [18] and the altered glycosylation profile of the rest of serum proteins in IgG-depleted sera from HT patients [19]. These structural studies are the only ones that together show the whole serum glycome in HT. Besides our studies, anti-Tg glycosylation in HT donors was analyzed by Yuan and coworkers, who showed higher content of sialic acids, mannose, core fucose and Gal(β1,4)GlcNAc(β1,2)Man structures on anti-Tg from HT as compared to anti-Tg from healthy donors. The enhanced sialylation and fucosylation of anti-Tg were correlated with the serum level of this antibody in HT [22]. These alterations of IgG glycosylation in HT prompted us to undertake the functional study and to develop the in vitro model to verify the hypothesis that altered IgG sialylation influences its effector function in thyroid autoimmunity. The present study used the whole pool of IgG instead of anti-TPO, known as the main inductor of ADCC and CDC in HT. The concentration of anti-TPO in serum is estimated to be up to 1.4 mg/mL [23], and the titer of anti-TPO in healthy donor sera was drastically lower than in HT (Table 1). As the result, it was impossible to isolate enough anti-TPO from healthy volunteer sera for the functional assays. Moreover, our previous results on altered glycosylation in AITD were obtained for the whole pool of IgG [18]. The results described in the Introduction section show that serum or IgG isolated from AITD patients is more potent in inducing cell lysis in ADCC and CDC in vitro models. Our present work confirmed these results and for the first time demonstrated the impact of IgG glycosylation, altered in HT, in thyrocyte lysis.

Due to the use of a relatively non-invasive method of serum collection, analyses of IgG glycosylation became a popular tool in the search for glyco-biomarkers of chronic inflammatory diseases [24,25]. Studies of in vitro models showed that genetic or enzymatic rearrangements of IgG *N*-glycans change its activity [26,27]. The vast majority of functional studies examined recombinant IgG molecules. The use of IgGs from HT and healthy donors with glycosylation altered during the autoimmune process gives our model a great advantage: it better reflects the pathological condition in the thyroid of HT patients, and the obtained results should serve as solid ground for further research.

Besides anti-TPO, the most common antibody present in over 90% of HT patients [28], other cell membrane proteins can induce an autoimmune response (with lower incidence), such as sodium/iodide symporter (NIS) and pendrin [29]. Anti-NIS and anti-pendrin IgGs were detected in 17% to 31% and 9% to 11% of AITD patients, respectively [30]. Anti-thyroid IgGs other than anti-TPO, present in the whole pool of IgG used in our model, can contribute to the cytotoxicity effect. On the other hand, other non-autoantigenic IgGs present in the whole pool of isolated IgG might also affect the results of our experiments. Metcalfe and coworkers showed significantly lower cell lysis in the presence of HT than in the presence of GD sera, but there was no correlation between anti-TPO serum level and thyrocyte lysis, which could result from the activity of other anti-thyroid IgGs in ADCC and autoantibodies against thyroid antigens not characteristic only of AITD [5]. To avoid the variability resulting from the presence of anti-thyroid IgGs other than anti-TPO in HT sera, we pooled the serum samples to each ADCC and CDC experiment and used the same amount of IgG per well.

The functional assays performed on ADCC and CDC models confirmed that IgGs isolated from HT patients induce thyroid cell lysis more efficiently (Figure 4). This significant increase of thyrocyte lysis resulted primarily from the higher titer of anti-TPO in HT serum samples (Table 1), despite having equal amounts of the whole pool of IgG in the HT and control variants used in both types of experiments. The statistically significant increase of thyroid cell lysis triggered by IgG from HT was observed only for HL-60 effector cells, not for PBMC. According to Rebuffat and coworkers, PBMC and unstimulated HL-60 cells express FcγRs (CD16, CD32, CD64) [7], responsible for their effector function. The diverse cytotoxic effects caused by PBMC and HL-60 may result from the difference in the heterogeneity of these two cell populations. HL-60 is a homogenous cell population, in contrast to the diverse phenotype of PBMC. Although PBMC is a natural source of effector cells, the heterogeneity of this population may mask the effect of cell activation.

The functional study with Neu-treated IgG, designed to assess the contribution of IgG glycosylation to thyrocyte lysis, showed that desialylated IgGs from HT donors are more potent than control IgG without SA in inducing the ADCC reaction, while in the CDC model, we observed lower lysis of thyroid cells triggered by desialylated IgG from HT than in control samples. The opposite effects on thyrocyte lysis in the ADCC and CDC models with the use of desialylated IgG might result from a difference in Fc IgG binding to the C1q complement component or Fc receptor (FcR), the initial stage of CDC and ADCC, respectively (Figure 5). Differences in effects in ADCC and CDC were also found by Quast and coworkers for sialylated therapeutic antibodies. Chemoenzymatic remodeling of *N*-glycosylation on Rituximab (RTX), a commercial monoclonal antibody (mAb) that recognizes CD20 antigen on B cells, led to obtaining fully sialylated IgG. Neither the sialylated RTX nor the unsialylated glycoform of this mAb influenced B cell lysis and FcR binding efficiency in the ADCC model. On the other hand, sialylated RTX reduced the intensity of CDC. This impairment of effector function by sialylated RTX was associated with a decrease of Fc binding to C1q, which initiates the classical complement pathway [31]. RTX was also used to determine an impact of Fc galactosylation on CDC. Galactosylation of IgG1 and IgG3 subclasses enhanced specific target cell lysis as a result of increased IgG binding to C1q, while the efficiency of galactosylated RTX in binding FcγRIIIa, necessary to activate the ADCC reaction, was unaffected [32]. Opposite effects of mAb glycosylation on ADCC and CDC were also shown in the case of Fc *N*-glycans with bisecting *N*-acetylglucosamine (GlcNAc). An increase of ADCC activity and a minimal effect on CDC were observed after modification of RTX and Herceptin *N*-glycans by adding bisecting GlcNAc. CDC was reduced by half with degalactosylated RTX in comparison to untreated IgG [33]. Functional effects of *N*-glycosylation remodeling on therapeutic mAb are well described, but the role of naturally occurring IgG with altered glycosylation in autoimmunity processes remains poorly understood.

To examine the impact of IgG glycosylation on its effector function, we compared the effect of thyrocyte lysis triggered by Neu-treated IgG to cell death induced by untreated IgG. The same enzymatic modification was applied in the ADCC assay to Pertuzumab, a therapeutic mAb, which recognizes human epidermal growth factor receptor 2 (HER2). A sixfold higher cytotoxic effect was observed in the case of sialylated and defucosylated Pertuzumab, and a 20-fold higher effect of cell lysis after desialylation and defucosylation of this mAb [34]. These results showed that the effect of IgG desialylation strictly depends on the presence of core fucose. Enzymatic removal of core fucose from *N*-glycans on native proteins is ineffective, because of the difficult access of fucosidase to a glycosidic bond linking fucose with an asparagine-linked GlcNAc in folded protein. In effect, the thyrocyte lysis in our models may have been influenced not only by a lack of IgG sialic acids but also by the presence of other components of IgG *N*-glycans. It has been estimated that over 90% of serum IgG glycoforms are fucosylated; in the fucosylated IgG pool, three galactosylated groups can be distinguished in terms of the number of galactose residues: agalactosylated (~35%), monogalactosylated (~35%), and digalactosylated (~16%). The minority of IgGs are sialylated, only 5% to 10% of antibodies have a single SA residue, and under 1% are disialylated [8,35]. Although the percentage of sialylated IgG is relatively low, the role of IgG sialylation is crucial in the effector function [8,36]. The lack of terminal SA enhances IgG binding to FcR and ADCC intensity [37]. The anti-inflammatory properties of sialylated IgG arise from an increased ability to bind DC-SIGN C-type lectin, which initiates inhibition of immune responses [38]. In our study, enzymatic removal of SA from serum IgGs made the IgG pool uniform in terms of sialylation and exposed the terminal galactose residues. A decrease of IgG galactosylation has been observed in different autoimmune and inflammatory diseases; among them, the significance of this modification is best known in rheumatoid arthritis [39,40]. Attenuation of IgG galactosylation is associated with the reduction of antibody immunosuppressive potential [41]. Besides exposure of Gal on IgG *N*-glycans, the observed enhancement of the cytotoxic effect in our study may have resulted from the decrease of IgG core fucosylation in AITD patients, described previously by Martin and coworkers [18].

## 5. Conclusions

Our results obtained from in vitro models of ADCC and CDC show the functional effect of altered sialylation of IgG isolated from HT patients. Removal of sialic acids from IgG *N*-glycans, accompanied by exposure of galactose residues, enhanced ADCC intensity and decreased the CDC process. Based on the results, we conclude that (1) IgGs isolated from Hashimoto’s thyroiditis patients induce a higher cytotoxic effect, and (2) altered sialylation of IgG affects thyrocyte lysis in both cytotoxicity models. Further research should determine the role of glycosylation in thyroid autoimmunity. We demonstrated the influence of altered glycosylation of IgG on thyroid cell lysis in the autoimmune condition. To investigate more comprehensively the role of glycosylation in HT pathogenesis, the impact of glycans present on effector cell receptors and complement proteins needs to be examined in detail.

## Figures and Tables

**Table 1:** Characteristics of Hashimoto’s thyroiditis (HT) and control (C) groups. Data in columns 3–7 are expressed as means ± SD (range). TgAb, thyroglobulin; TPOAb, anti-thyroperoxidase; TRAb, anti-thyrotropin-releasing hormone; TSH, thyroid-stimulating hormone.

Group	Sex (M/F)	Age Mean ± SD (Range)	TSH 0.27–4.20 uIU/mL	TPOAb <34.0 IU/mL	TgAb <115.0 IU/mL	TRAb 0.0–1.0 IU/mL
C (*n* = 24)	0/24	34.9 ± 6.5 (23–46)	2.7 ± 1.3 (0.5–4.2)	14.1 ± 6.7 (8.5–33.0)	15.3 ± 16.0 (10.0–89.3)	0.3 ± 0.3 (0.1–1.0)
HT (*n* = 24)	0/24	33.6 ± 7.0 (24–49)	2.5 ± 1.2 (0.2–4.9)	229.2 ± 148.2 (43.0–542.0)	291.7 ± 230.8 (13.8–873.1)	0.6 ± 0.3 (0.1–1.1)
